# Association of Consuming Tap Water or Purified Water during Infancy with Irritable Bowel Syndrome in Children

**DOI:** 10.3390/children9020135

**Published:** 2022-01-20

**Authors:** Ju Hee Kim, Hey-Sung Baek, Eun Kyo Ha, Hye Ryung Cha, Seung Won Lee, Man Yong Han

**Affiliations:** 1 Department of Pediatrics, Kangdong Sacred Heart Hospital, Hallym University College of Medicine, Seoul 05355, Korea; 210150@kdh.or.kr (J.H.K.); paviola7@kdh.or.kr (H.-S.B.); 2 Department of Pediatrics, Kangnam Sacred Heart Hospital, Hallym University College of Medicine, Seoul 07441, Korea; dmsry1@hallym.or.kr; 3 Department of Data Science, Sejong University College of Software Convergence, Seoul 05006, Korea; ryoung0156@gmail.com; 4 Department of Precision Medicine, Sungkyunkwan University School of Medicine, Suwon 16419, Korea; 5 Department of Pediatrics, Bundang CHA Medical Center, CHA University School of Medicine, Seongnam 13496, Korea

**Keywords:** tap water, purified water, gut microbiota, irritable bowel syndrome

## Abstract

Objective: The objective of this study was to analyze the effect of consuming formula powder prepared with tap water or purified water during the first 4 to 6 months of life on the subsequent development of irritable bowel syndrome (IBS). Study design and setting: A total of 917,707 children who were born in Korea between 2007 and 2008 were analyzed. All children were followed up until they lost eligibility for health care services or until 2017. Data on the water used to prepare formula powder were from questionnaires answered by the parents when the child was 4 to 6 months old. IBS was defined as two or more diagnoses of IBS after the age of 4 years. Inverse probability of treatment weighting (IPTW) using the propensity score was used to balance the two groups. The risk of IBS was evaluated using a Cox proportional hazards model. Results: After weighting, there were 73,355 children in the tap water group and 73,351 in the purified water group. The purified water group had a higher risk of IBS (HR: 1.05; 95% CI: 1.01, 1.09). This relationship was also present after the subgroup analyses of males and females and the sensitivity analysis that used different definitions of IBS. Conclusions: Drinking formula powder prepared with purified water rather than tap water during the first 4 to 6 months of age was found to be associated with IBS.

## 1. Introduction

Water is not only the first substance consumed by a baby after birth, but also a major component of the food eaten by infants. Many studies have reported associations between poor drinking water and human health, especially contaminated drinking water with infectious diseases [1,2]. However, developed countries have fewer concerns about contaminated drinking water due to the high quality of household water [3].

A newborn’s gut microbiota is determined by contact with the mother and the environment, especially food intake [4]. In particular, the first 6 months of life is considered a critical period, during which environmental factors affect microbial colonization [5,6]. The development of gut microbiota during early life is related to health status later in life. During their first 6 months, babies consume only a limited variety of foods, mainly breast milk and/or formula powder. Different types of water may be used to prepare formula powder. There is a limited understanding of the effect of consuming formula powder made with different types of water on the gut microbiota of babies. However, a previous study reported that the bacteria in drinking water affected the establishment of gut microbiota in germ-free mice [7]. Another animal study also showed that different types of drinking water led to differences in gut microbiota [8].

Therefore, we analyzed the effects of consuming formula powder prepared with different types of drinking water (tap water vs. purified water) during the first 4 to 6 months of life on the subsequent development of irritable bowel syndrome (IBS), a significant disease that is affected by the composition of gut microbiota.

## 2. Methods

### 2.1. Study Design and Setting

This study used the National Investigation of Birth Cohort in Korea (NICKs-2008) dataset to examine 917,707 children who were born in Korea between 2008 and 2009 [9]. The National Health Screening Program for Infants and Children (NHSPIC) is a surveillance program provided to all health insurance subscribers from the age of 4 to 72 months. The National Health Insurance Service (NHIS) is the health insurance system that covers nearly the entire population of South Korea and provides data about healthcare utilization. These data include International Classification of Disease 10th Version (ICD-10) codes, duration of hospital admission, treatment costs, physician services provided, and prescriptions (including drug classification codes and days prescribed). The Checklist of Recommendations based on REporting of studies Conducted using Observational Routinely-collected health Data is described in Table S1. The use of de-identified individual data for research purposes was authorized under the current National Health Insurance Act. The protocol of this study was reviewed and approved by the Institutional Review Board of the Korea National Institute for Bioethics Policy (P01-201603-21-005, date: 4 October 2018).

### 2.2. Data Sources

The dataset from NICK-2008, acquired from the NHIS and NHSPIC database, was used to analyze multiple variables (Table S2). Health care utilization data were recorded during the process of making claims for healthcare services. Residential status at birth was classified as Seoul, metropolitan (Busan, Daegu, Incheon, Gwangju, Daejeon, and Ulsan), urban, or rural. Socio-economic status was classified by quintile, according to the amount of insurance co-payment. All participants were followed until the loss of eligibility for health care services, death, immigration, or the end of 2017.

### 2.3. Participants

**Figure 1:**
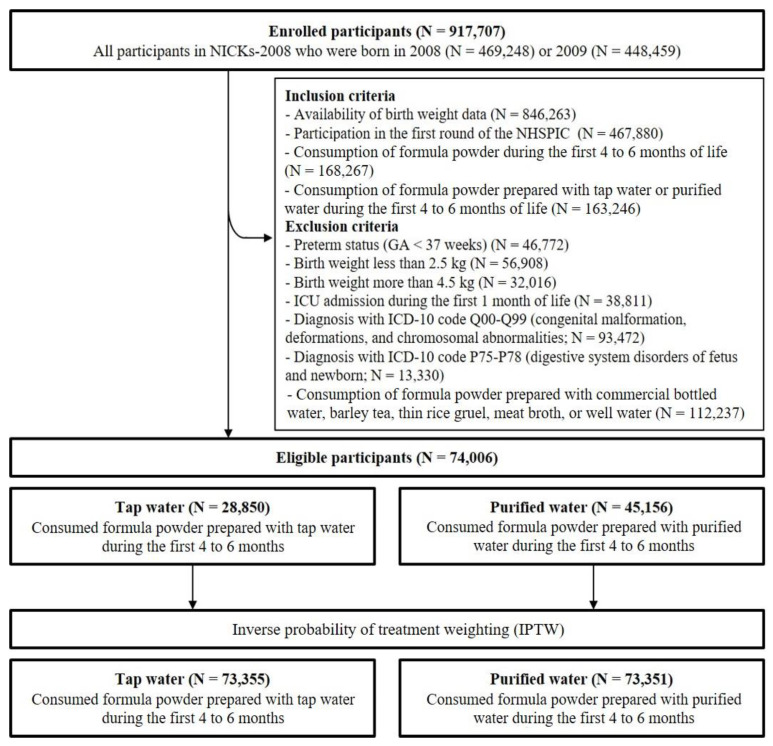
Enrollment, assessment of eligibility, and weighting of study participants. Abbreviations: N, number; NICKs-2008, the National Investigation of Birth Cohort in Korea; ICU, intensive care unit; ICD, International Classification of Disease.

The cohort consisted of children whose parents responded to the questionnaire when they were 4 to 6 months old and had recorded birth weights (Figure 1). It has already been reported that breastfeeding in early infancy decreases the risk of irritable bowel syndrome compared to formula-feeding [10]. As our purpose is to compare the associations between irritable bowel syndrome and consuming tap water or purified water in early infancy, we enrolled only children who consumed formula powder that was prepared with tap water or purified water during the first 4 to 6 months of life. Children were excluded if they were born before 37 weeks of gestational age, had a birth weight less than 2.5 kg or more than 4.5 kg, were admitted to an intensive care unit within the first month, or were diagnosed with a congenital malformation, deformation, chromosomal abnormality, or digestive system disorder as a fetus or newborn. Children were also excluded if they consumed breast milk, partially consumed formula powder, or mainly drank formula powder prepared with commercial bottled water, barley tea, thin rice gruel, meat broth, or well water. A total of 74,006 children met the inclusion and exclusion criteria. Among these children, 28,850 children who mainly drank formula powder prepared with tap water were allocated to a tap water group and 45,156 children who mainly drank formula powder prepared with purified water were allocated to a purified water group (Figure 1).

### 2.4. Exposure

The exposure of interest was the consumption of formula powder prepared using tap water or purified water during the first 4 to 6 months of life. Questions about the main type of water were obtained using a questionnaire answered by the parents when the child was 4 to 6 months old. This question was as follows: “What kind of water did you use to mix formula powder?” The possible answers were commercial bottled water, tap water, purified water, barley tea, thin rice gruel/meat broth, or well water. We defined the tap water group to be the reference group. Purified water was defined as water that had passed through a water purifier, regardless of the type of filter used in the water purifier.

### 2.5. Outcomes

The main outcome was the risk of IBS after the age of 48 months. IBS was defined as more than two diagnoses with an ICD-10 code of K58.X (IBS) after the age of 4 years [11]. The number of subjects with IBS in the cohort was 22,918 (15.6%), which is consistent with the NICKs-2008 cohort (917,707, 15.2%) (Table S3).

To assess the robustness of the results, sensitivity analyses were performed using different definitions of IBS. IBS-1 was defined as at least one diagnostic ICD-10 code of K58.X (IBS) and R10.X (abdominal pain), and at least one ICD-10 code of K59.1 (functional diarrhea), K52.2 (allergic and dietetic gastroenteritis and colitis), K52.8 (other specific noninfective gastroenteritis), or K59.0 (constipation) after the age of 4 years; a previous study showed that this definition had a positive predictive value (PPV) of 83% (95% CI: 75%, 91%) [12]. IBS-2 was defined as two or more ICD-10 codes of K58.X (IBS) that were more than 6 months apart, a definition that had a PPV of 75% (95% CI: 66%, 84%) [12].

### 2.6. Covariates

A total of 103 covariates were used to balance the two groups (Table S2). These included multiple demographic variables (age, sex, income quintile, and residence at birth) and comorbidities. The comorbidities were identified from head-to-extremity general physical examinations provided by physicians during the first round of the NHSPIC. Perinatal conditions were evaluated using ICD-10 codes. To evaluate clinical conditions within the first six months of age, the most prevalent 47 diseases, including respiratory infections, gastrointestinal infections, and dermatitis, were identified using the ICD-10 codes. In addition, 18 types of drugs, including drugs commonly administered to infants, were defined using drug classification codes. Hospitalization records, emergency room visits, and visits to pediatricians within the first six months of life were also recorded.

### 2.7. Statistical Analysis

An inverse probability of treatment weighting (IPTW) using the propensity score was used to balance the two groups for the 103 a priori covariates (Table S2). Each propensity score was estimated using a multivariable logistic regression. The tap water group (reference group) was weighted as (propensity score)/(1 − propensity score) [13]. This produced a weighted pseudo-sample of patients in the reference group with the same distribution of measured covariates as in the exposure group [14]. A comparison of between-group differences in baseline characteristics was used to determine standardized differences in the unweighted and weighted samples, and a difference greater than 10% was considered meaningful [15]. A Cox proportional hazards model was used to assess the risk of IBS according to the type of water consumed. Hazard ratios (HRs) and 95% CIs were calculated for each outcome of interest. Post hoc sensitivity analyses, analyses using a negative control exposure (defined as commercial bottled water), were used to assess the robustness of the main results. Two-tailed *p*-values less than 0.05 were interpreted as significant. All statistical analyses were performed using SAS version 9.4 (SAS Institute Inc., Cary, NC, USA).

## 3. Results

### 3.1. Participants

After IPTW, we allocated 73,355 children to the tap water group and 73,351 children to the purified water group (Figure 1). We first compared the basic sociodemographic characteristics of these two groups before and after weighting (Table 1). The weighted data indicated these two groups were balanced for all variables. These two groups were also balanced for all variables from physical examinations and questionnaires, including visual and auditory senses at the age of 4 to 6 months (Table S4).

We then examined the basic clinical characteristics of the two groups (Table 2). The analysis of hospital utilization and perinatal conditions indicated the two groups were balanced. The analysis of the prevalence of diseases diagnosed within the first six months of age and the use of major drugs also indicated these groups were balanced, with all standardized differences less than 10% after weighting. The analysis of the full set of clinical characteristics also indicated the groups were balanced in all analyzed variables (Table S5, Supplementary Materials).

### 3.2. Association of the Type of Drinking Water with a Diagnosis of IBS after 4 Years of Age

We then determined the association of the type of water used to prepare formula powder with a diagnosis of IBS (Table 3). In the weighted data, there were 11,197 (15.3%) children with IBS in the tap water group and 11,721 (16.0%) in the purified water group, corresponding to an HR of 1.051 (95% CI: 1.013, 1.090; *p*-value < 0.01).

### 3.3. Sensitivity Analysis

**Figure 2:**
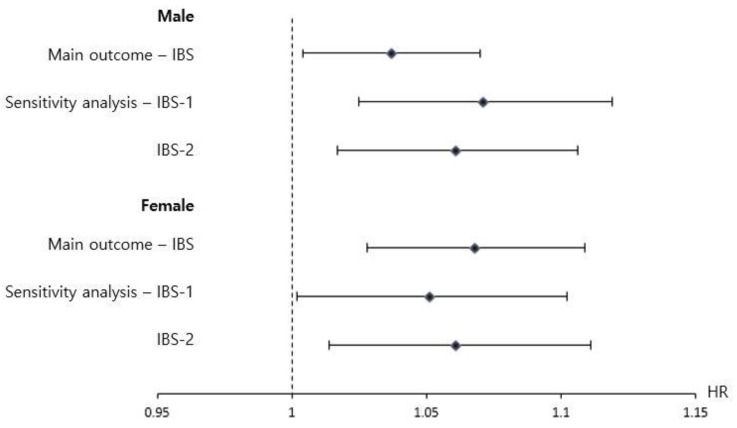
Subgroup analysis (with stratification by sex) and sensitivity analysis (using different definitions of IBS) of the risk of IBS in children at the age of 4 to 6 years who consumed formula powder prepared with purified water rather than tap water (reference) during infancy. Irritable bowel syndrome as the main outcome was defined as more than twice the diagnosis of ICD-10 codes K58.X (irritable bowel syndrome) after 4 years of age. IBS-1 was defined as at least one diagnosis of ICD-10 code K58.X (IBS) and R10.X (abdominal pain) and at least one of ICD-10 code K59.1 (functional diarrhea), K52.2 (allergic and dietetic gastroenteritis and colitis), K52.8 (other specific noninfective gastroenteritis), or K59.0 (constipation) after the age of four years. IBS-2 was defined as two or more diagnoses of ICD-10 code K58.X (irritable bowel syndrome) with an interval of more than 6 months. This method produces a weighted pseudo-sample of participants in the reference group with the same distribution of measured covariates as the exposure group. Filled squares indicate hazard ratios, and straight lines indicate 95% confidence intervals. The asterisks indicate *p* < 0.05. The hazard ratios were assessed using a Cox proportional hazards model to examine the risk of IBS development in the cohort.

The sensitivity analysis, which used two alternate definitions for IBS (IBS-1 and IBS-2), indicated the purified water group had an increased risk of IBS-1 (HR: 1.062; 95% CI: 1.014, 1.111; *p*-value = 0.01) and IBS-2 (HR: 1.061; 95% CI: 1.016, 1.108; *p*-value < 0.01). In addition, separate analyses of males and females indicated that the risk of IBS, IBS-1, and IBS-2 remained statistically significant (Figure 2).

### 3.4. Post Hoc Analysis: Tap Water vs. Commercial Bottled Water

We also performed a post hoc analysis to compare children who consumed commercial formula powder prepared with bottled water or tap water (Table S6). As in the main analysis, these two groups were matched using IPTW for all variables (Table S2). The results of the weighted analysis indicated 7490 (15.3%) children with IBS were in the tap water group and 7526 (15.4%) were in the commercial bottled water group. The risk of IBS in these two groups was not statistically significant (HR: 1.004; 95% CI: 0.960, 1.051; *p*-value = 0.86).

## 4. Discussion

Our large national administrative cohort study showed that the use of purified water rather than tap water to prepare formula powder for young infants was associated with the subsequent development of IBS. More specifically, formula-fed children who mainly consumed formula powder prepared with purified water during their first 4 to 6 months of life had a negative association with the development of IBS after the age of 4 years. Furthermore, our sensitivity analyses indicated that this association remained significant when using different definitions of IBS, and our subgroup analyses indicated this association was significant for males and females. However, we found that the consumption of commercial bottled water rather than tap water had no impact on the risk of IBS.

It is well known that an aberrant gut microbiota can contribute to the development of IBS [16,17]. Because drinking water that is safe to consume still contains microbes, our findings suggest the type of water used to prepare formula powder may have affected the gut microbiota of infants. Although the various treatment processes and chlorination have greatly improved the quality of tap water, the microbes present in high-quality treated tap water can vary among regions. A study of germ-free mice reported that the microbiota of their gastrointestinal tracts were similar to those of other mice due to the consumption of microorganisms in the drinking water [7]. In addition, the type of drinking water can affect the gut microbiota composition. In particular, mice that consumed autoclaved water had a more homogeneous gut microbiota than those that consumed tap water or bottled mineral water, presumably because of exposure to water-borne bacteria [18]. In contrast, most water purifiers introduced into South Korea during 2008 and 2009 use reverse osmosis to filter out impurities, minerals, and bacteria. The diversity of gut microbiota is significantly lower in IBS patients than in healthy individuals. This suggests that drinking purified water during the first 6 months of age—a critical period for the establishment of gut microbiota—had an adverse effect on the diversity of gut microbiota and contributed to the development of IBS.

To the best of our knowledge, this study is the first to compare the risk of IBS due to the consumption of formula powder prepared with tap water or purified water. A strength of our study is that we examined all formula-fed children born in Korea between 2008 and 2009. However, there were also some limitations in our study. First, our definition of IBS was based on ICD-10 codes from an administrative database. However, we addressed this limitation by performing identical analyses with two different validated definitions of IBS that had a high diagnostic yield in previous studies [11,12]. Second, it is well known that the risk of IBS is greater in those with a higher socioeconomic status [19] and with psychogenic problems [20], such as anxiety and depression [21]. Although we attempted to match the two groups with respect to health-related and demographic variables, there may have been some selection bias, so our results cannot be used to infer causal relationships. Third, there was a lack of information about whether the tap water was boiled or not and the type of filter of the water purifier. Lastly, because the associations between purified water and IBS that we found are slightly small, further research is needed to confirm the association between them.

## 5. Conclusions

Consuming formula powder prepared from purified water rather than tap water during the first 4 to 6 months of age has a potential risk for the development of IBS at the age of 4 to 6 years. This study provides new insights regarding the association between human health and the type of water consumed during early infancy. Further studies are needed to confirm our findings and to identify the underlying pathogenic mechanism of this association.

## Figures and Tables

**Table 1:** Basic sociodemographic characteristics of the participants ^a^.

Sociodemographic Characteristics	Observed Data (N = 74,006)			Weighted Data (N = 146,706) ^b^		
	N (%) ^c^		Standardized Difference (%) ^f^	N (%) ^c^		Standardized Difference (%) ^d^
	Tap Water ^d^	Purified Water ^e^		Tap Water ^d^	Purified Water ^e^	
	(N = 28,850)	(N = 45,156)		(N = 73,355)	(N = 73,351)	
Sex						
Male	15,156 (52.5)	23,704 (52.5)	0.1	38,532 (52.5)	38,506 (52.5)	0.1
Female	13,694 (47.5)	21,452 (47.5)		34,824 (47.5)	34,845 (47.5)	
Residence at birth ^g^						
Seoul	6625 (23.0)	10,615 (23.5)	1.3	17,115 (23.3)	17,089 (23.3)	0.0
Metropolitan	6732 (23.3)	11,165 (24.7)	3.3	17,715 (24.2)	17,737 (24.2)	0.1
Urban	11,614 (40.3)	18,361 (40.7)	0.8	29,715 (40.5)	29,712 (40.5)	0.1
Rural	3619 (12.5)	4620 (10.2)	7.3	8162 (11.1)	8164 (11.1)	0.0
Birth weight, kg (SD) ^h^	3.22 (0.32)	3.22 (0.32)	1.5	3.22 (0.51)	3.22 (0.41)	0.0
Income quintile ^i^						
1 (Lowest)	2300 (8.0)	3384 (7.5)	1.8	5643 (7.7)	5636 (7.7)	0.0
2	4557 (15.8)	6177 (13.7)	6.0	10,628 (14.5)	10,632 (14.5)	0.0
3 (Middle)	8215 (28.5)	11,238 (24.5)	8.1	19,316 (26.3)	19,305 (26.3)	0.0
4	9141 (31.7)	14,374 (31.8)	0.3	23,287 (31.7)	23,297 (31.8)	0.0
5 (Highest)	3757 (13.0)	8399 (18.6)	15.3	12,044 (16.4)	12,042 (16.4)	0.0
Birth year						
2008	13,140 (45.5)	21,566 (47.8)	4.4	34,291 (46.7)	34,318 (46.8)	0.1
2009	15,710 (54.5)	23,590 (52.2)		39,064 (53.3)	39,033 (53.2)	

Abbreviations: N, Number; SD, standard deviation. ^a^ Unless otherwise specified, baseline characteristics were assessed on the birth date of the patient. ^b^ Weighted using inverse probability of exposure weighting based on the propensity score. The propensity score was estimated using multivariable logistic regression with 103 previous covariates, as defined in Table S2 in the Supplementary Materials. Participants in the reference group were weighted as (propensity score)/(1 − propensity score). This method produces a weighted pseudo-sample of participants in the reference group with the same distribution of measured covariates as the exposure group. ^c^ Results are reported as N (%) unless otherwise indicated. ^d^ The tap water (reference) group consisted of children who mainly consumed formula powder prepared with tap water during the first 4 to 6 months of life. ^e^ The purified water group consisted of children who mainly consumed formula powder prepared with purified water during the first 4 to 6 months of life. ^f^ Differences greater than 10% were interpreted as meaningful differences. All standardized differences in the cohort values were <0.05. ^g^ Metropolitan areas were defined as six metropolitan cities (Busan, Incheon, Gwangju, Daejeon, Daegu, and Ulsan), urban areas as cities, and rural areas as non-city areas. Missing data of observed data: tap water = 260, purified water = 395; missing data of weighted data: tap water = 646, purified water = 649. ^h^ Obtained from the 1st National Health Screening Program of Infants and Children at 4–6 months of birth. ^i^ Income status was categorized into the quintile of insurance premium at birth. Missing data of observed data: tap water = 880, purified water = 1584; missing data of weighted data: tap water = 2438, purified water = 2440.

**Table 2:** Basic clinical characteristics of the participants ^a^.

Clinical Characteristics	Observed Data (N = 74,006)			Weighted Data (N = 146,706) ^b^		
	N (%) ^c^		Standardized Difference (%) ^f^	N (%) ^c^		Standardized Difference (%) ^f^
	Tap Water ^d^	Purified Water ^e^		Tap Water ^d^	Purified Water ^e^	
	(N = 28,850)	(N = 45,156)		(N = 73,355)	(N = 73,351)	
Hospital utilization within six months of age						
Visiting to pediatricians	28,178 (97.7)	44,116 (97.7)	0.2	71,666 (97.7)	71,658 (97.7)	0.0
Visiting ER	2281 (7.9)	3789 (8.4)	1.7	6030 (8.2)	6010 (8.2)	0.0
Hospitalization	5150 (17.9)	8419 (18.6)	2.1	13,452 (18.3)	13,458 (18.3)	0.0
Certain conditions (ICD-10 codes) originating in the perinatal period						
Respiratory and cardiovascular disorders specific to the perinatal period	1199 (4.2)	1793 (4.0)	0.9	2974 (4.1)	2967 (4.1)	0.0
Infections specific to the perinatal period	4066 (14.1)	6176 (13.7)	1.2	10,152 (13.8)	10,146 (13.8)	0.0
Hemorrhagic and hematological disorders of fetuses and newborns	8051 (27.9)	12,608 (27.9)	0.0	20,483 (27.9)	20,484 (27.9)	0.0
Prevalent diseases (ICD-10 codes) diagnosed within six months of age						
Viral intestinal infection, unspecified	9741 (2.7)	459 (5.2)	12.9	10,064 (2.8)	11,772 (3.3)	0.6
Other and unspecified gastroenteritis and colitis of infectious origin	18,362 (5.1)	707 (8.0)	11.9	18,802 (5.2)	20,941 (5.8)	0.7
Gastroenteritis and colitis of unspecified origin	20,055 (5.6)	781 (8.9)	12.8	20,541 (5.7)	22,505 (6.3)	0.8
Conjunctivitis, unspecified	14,776 (4.1)	397 (4.5)	2.0	14,961 (4.1)	14,662 (4.1)	0.4
Otitis media, unspecified	7989 (2.2)	204 (2.3)	0.7	8058 (2.2)	7869 (2.2)	0.2
Acute nasopharyngitis	104,381 (29.0)	3,056 (34.8)	12.4	105,974 (29.2)	107,273 (29.8)	0.1
Acute sinusitis, unspecified	12,585 (3.5)	304 (3.5)	0.2	12,707 (3.5)	12,261 (3.4)	0.4
Acute pharyngitis	37,920 (10.5)	1103 (12.6)	6.3	38,483 (10.6)	39,901 (11.1)	0.6
Acute tonsillitis, unspecified	18,232 (5.1)	464 (5.3)	1.0	18,411 (5.1)	18,064 (5.0)	0.1
Other acute upper respiratory infections of multiple sites	9292 (2.6)	265 (3.0)	2.6	9427 (2.6)	9916 (2.8)	0.3
Acute upper respiratory infection, unspecified	81,665 (22.7)	2198 (25.0)	5.4	82,711 (22.8)	84,377 (23.4)	0.5
Pneumonia, unspecified	9496 (2.6)	225 (2.6)	0.5	9569 (2.6)	10,044 (2.8)	0.6
Acute bronchitis	66,304 (18.4)	1,634 (18.6)	0.4	66,995 (18.4)	66,559 (18.5)	0.7
Acute bronchiolitis	48,798 (13.6)	1,129 (12.8)	2.1	49,176 (13.5)	50,601 (14.1)	0.7
Gastro-esophageal reflux disease without esophagitis	8416 (2.3)	333 (3.8)	8.4	8617 (2.4)	8590 (2.3)	0.4
Functional dyspepsia	7635 (2.1)	315 (3.6)	8.8	7834 (2.2)	8289 (5.1)	0.2
Noninfective gastroenteritis and colitis, unspecified	16,372 (4.6)	561 (6.4)	8.1	16,709 (4.6)	18,306 (5.1)	0.8
Constipation	15,365 (4.3)	574 (6.5)	10.0	15,699 (4.3)	16,272 (4.5)	0.7
Functional diarrhea	5223 (1.5)	205 (2.3)	6.5	5360 (1.5)	5375 (1.5)	0.0
Functional intestinal disorder, unspecified	10,060 (2.8)	357 (4.1)	7.0	10,248 (2.8)	10,274 (2.9)	0.1
Nausea and vomiting	10,334 (2.9)	495 (5.6)	13.7	10,699 (2.9)	11,869 (3.3)	0.5
Fever, unspecified	18,237 (5.1)	524 (6.0)	3.9	18,507 (5.1)	21,194 (5.9)	0.1
Drug (drug classification code) used within six months of age						
Antipyretics	17,166 (59.5)	28,027 (62.1)	5.3	44,808 (61.1)	44,796 (61.1)	0.0
Antihistamine	19,707 (68.3)	32,541 (72.1)	8.2	51,843 (70.7)	51,817 (70.6)	0.0
Digestive anti-ulcer drug	524 (1.8)	884 (2.0)	1.0	1384 (1.9)	1390 (1.9)	0.0
Antiacid drug	313 (1.1)	558 (1.2)	1.4	858 (1.2)	861 (1.2)	0.0
Probiotics	20,061 (69.5)	32,396 (71.7)	4.8	51,976 (70.9)	51,983 (70.2)	0.0
Laxative drug	39 (0.1)	76 (0.2)	0.9	114 (0.2)	114 (0.2)	0.0
Other digestive system drugs	11,080 (38.4)	17,939 (39.7)	2.7	28,760 (39.2)	28,760 (39.2)	0.0
Steroid	3481 (12.1)	6,199 (13.7)	5.0	9588 (13.1)	9592 (13.1)	0.0

Abbreviations: N, Number; ICD, International Classification of Diseases; ER, emergency room; ICU, intensive care unit. ^a^ Unless otherwise specified, all baseline characteristics were assessed in the first six months of age of the participants. ^b^ Weighted using inverse probability of exposure weighting based on the propensity score. The propensity score was estimated using multivariable logistic regression with 103 previous covariates, as defined in Table S2 in the Supplementary Materials. Participants in the reference group were weighted as (propensity score)/(1 − propensity score). This method produces a weighted pseudo-sample of participants in the reference group with the same distribution of measured covariates as the exposure group. ^c^ Results are reported as N (%) unless otherwise indicated. ^d^ The tap water (reference) group consisted of children who mainly consumed formula powder prepared with tap water during the first 4 to 6 months of life. ^e^ The purified water group consisted of children who mainly consumed formula powder prepared with purified water during the first 4 to 6 months of life. ^f^ Differences greater than 10% were interpreted as meaningful differences. All standardized differences in the cohort values were <0.05.

**Table 3:** Risk of irritable bowel syndrome ^a^.

	Observed Data (N = 74,006)		Weighted Data (N = 146,706) ^b^		HR (95% CI) ^e^	*p*-Value
	N (%)		N (%)			
	Tap water ^c^	Purified water ^d^	Tap water ^c^	Purified water ^d^		
	(N = 28,850)	(N = 45,156)	(N = 73,355)	(N = 73,351)		
Main outcome						
IBS ^f^	4380 (15.2)	7216 (16.0)	11,197 (15.3)	11,721 (16.0)	1.051 (1.013 to 1.090)	<0.01
Sensitivity analysis						
IBS-1 ^g^	2787 (9.66)	4686 (10.4)	7176 (9.8)	7598 (10.4)	1.062 (1.014 to 1.111)	0.01
IBS-2 ^h^	3059 (10.6)	5103 (11.3)	7827 (10.7)	8277 (11.3)	1.061 (1.016 to 1.108)	<0.01

Abbreviations: N, Number; IBS, irritable bowel syndrome; HR, hazard ratio; CI, confidence interval. ^a^ The cohort consisted of the infantile colic group who had experienced infantile colic from five weeks to four months of age and the control group without infantile colic histories during the same time period. ^b^ Weighted using inverse probability of exposure weighting based on the propensity score. The propensity score was estimated using multivariable logistic regression with 103 previous covariates, as defined in Table S2 in the Supplementary Materials. Participants in the reference group were weighted as (propensity score)/(1 − propensity score). This method produces a weighted pseudo-sample of participants in the reference group with the same distribution of measured covariates as the exposure group. ^c^ The tap water (reference) group consisted of children who mainly consumed formula powder prepared with tap water during the first 4 to 6 months of life. ^d^ The purified water group consisted of children who mainly consumed formula powder prepared with purified water during the first 4 to 6 months of life. ^e^ The hazard ratios were assessed using a Cox proportional hazards model to assess the risk of IBS according to the type of used water (tap water vs. purified water). ^f^ IBS was defined as more than two diagnoses with ICD-10 code K58.X (IBS) after the age of 4 years. ^g^ IBS-1 was defined as at least one diagnosis with ICD-10 code K58.X (IBS) and R10.X (abdominal pain), and an ICD-10 code K59.1 (functional diarrhea), K52.2 (allergic and dietetic gastroenteritis and colitis), K52.8 (other specific noninfective gastroenteritis), or K59.0 (constipation) after the age of four years. ^h^ IBS-2 was defined as two or more diagnoses of ICD-10 code K58.X (IBS) that were more than 6 months apart.

## Data Availability

This study was based on the National Health Claims Database (NHIS-2019-1-560) established by the NHIS of the Republic of Korea. If the application for using HNIS data is approved by the Inquiry Committee of Research Support, raw data is provided to the applicant for a fee. We cannot provide access to the data, analytic methods, and research materials to other researchers because of the intellectual property rights of this database that is owned by the National Health Insurance Corporation. However, investigators who wish to reproduce our results or replicate the procedure can be used in the database, which is open for research purposes (https://nhiss.nhis.or.kr/ (accessed on 13 November 2019)).

## Supplementary Materials

The following supporting information can be downloaded at: https://www.mdpi.com/article/10.3390/children9020135/s1, Table S1: Checklist of Recommendations for Reporting of Observational Studies Using the Reporting of Studies Conducted Using Observational Routinely Collected Health Data (RECORD) Guidelines; Table S2: Covariables in the propensity score matching; Table S3: Operating characteristics of the diagnostic codes used to define the diseases; Table S4: Results of the questionnaires and physical examinations of the first round of the NHSPIC in the cohort; Table S5. Clinical characteristics of the participants; Table S6. Post-hoc analysis using a negative control exposure to assess the risk of IBS between tap water and commercial bottled water.

## Abbreviations

IBS, irritable bowel syndrome; NHSPIC, National Health Screening Program for Infants and Children; NHIS, National Health Insurance Service; ICD-10, International Classification of Disease 10th Version; IPTW, inverse probability of treatment weighting; HR, hazard ratio.
