# Supplementary material for: Association of Consuming Tap Water or Purified Water during Infancy with Irritable Bowel Syndrome in Children
Source: Children (Basel). 2022 Jan 20;9(2):135. doi: 10.3390/children9020135 (PMC8869972; doi:10.3390/children9020135)
Supplement: Supplementary file 1 [file children-09-00135-s001.zip › children-1449417-supplementary.pdf]

Table S1. Checklist of Recommendations for Reporting of Observational Studies Using the Reporting of Studies Conducted Using Observational Routinely Collected Health Data (RECORD) Guidelines

	Item No	Recommendation	Reported
Title and abstract	1	(a) Indicate the study's design with a commonly used term in the title or the abstract	Abstract
		(b) Provide in the abstract an informative and balanced summary of what was done and what was found	Abstract
**Introduction**			
Background /rationale	2	Explain the scientific background and rationale for the investigation being reported	Introduction
Objectives	3	State specific objectives, including any prespecified hypotheses	Introduction
**Methods**			
Study design	4	Present key elements of study design early in the paper	Methods - Study Design and Setting
Setting	5	Describe the setting, locations, and relevant dates, including periods of recruitment, exposure, follow-up, and data collection	Methods - Study Design and Setting, Data sources
Participants	6	(a) Give the eligibility criteria, and the sources and methods of selection of participants. Describe methods of follow-up	Methods – Study population and Figure 1
		(b) For matched studies, give matching criteria and number of exposed and unexposed	Methods – Covariates, Statistical analysis; Results
Variables	7	Clearly define all outcomes, exposures, predictors, potential confounders, and effect modifiers. Give diagnostic criteria, if applicable	Methods - Data Sources; Exposure; Outcome; Covariates
Data sources /measurement	8	For each variable of interest, give sources of data and details of methods of assessment (measurement). Describe comparability of assessment methods if there is more than one group	Methods - Data Sources;
Bias	9	Describe any efforts to address potential sources of bias	Methods – Covariates; Statistical analysis;
Study size	10	Explain how the study size was arrived at	Methods – study population, Figure 1
Quantitative variables	11	Explain how quantitative variables were handled in the analyses. If applicable, describe which groupings were chosen and why	Methods – statistical analysis
Statistical methods	12	(a) Describe all statistical methods, including those used to control for confounding	Methods – Covariates; statistical analysis

		(b) Describe any methods used to examine subgroups and interactions	Methods – statistical analysis
		(c) Explain how missing data were addressed	Table 1
		(d) If applicable, explain how loss to follow-up was addressed	Not applicable
		(e) Describe any sensitivity analyses	Methods – Study population; Statistical analysis
**Results**			
Participants	13	(a) Report numbers of individuals at each stage of study, e.g., numbers potentially eligible, examined for eligibility, confirmed eligible, included in the study, completing follow-up, and analyzed	Results - Participants; Figure 1
		(b) Give reasons for non-participation at each stage	Figure 1
		(c) Consider use of a flow diagram	Figure 1
Descriptive data	14	(a) Give characteristics of study participants (e.g. demographic, clinical, social) and information on exposures and potential confounders	Results - Participants; Table 1, Table 2, eTable 4, and eTable 5
		(b) Indicate number of participants with missing data for each variable of interest	Methods – Table 1
		(c) Summarize follow-up time (e.g. average and total amount)	Methods – Data sources
Outcome data	15	Report numbers of outcome events or summary measures over time	Results – Table 3, Figure 2
Main results	16	(a) Give unadjusted estimates and, if applicable, confounder-adjusted estimates and their precision (e.g., 95% confidence interval). Make clear which confounders were adjusted for and why they were included	Results – eTable 2, Table 3, eTable 6
		(b) Report category boundaries when continuous variables were categorized	Not applicable
		(c) If relevant, consider translating estimates of relative risk into absolute risk for a meaningful time period	Not applicable
Other analyses	17	Report other analyses done, e.g., analyses of subgroups and interactions, and sensitivity analyses	Results – Table 3, eTable 6
**Discussion**			
Key result	18	Summarize key results with reference to study objectives	Discussion
Limitation	19	Discuss limitations of the study, taking into account sources of potential bias or imprecision. Discuss both direction and magnitude of any potential bias	Discussion

Interpretation	20	Give a cautious overall interpretation of results considering objectives, limitations, multiplicity of analyses, results from similar studies, and other relevant evidence	Discussion
Generalizability	21	Discuss the generalizability (external validity) of the study results	Discussion
Other information			
Funding	22	Give the source of funding and the role of the funders for the present study and, if applicable, for the original study on which the present article is based	Article Information

Table S2. Covariables in the propensity score matching

Characteristic	Database		Variables
**Demographics**	NHIS		Age, sex, income quintile, residence at birth
	NHSPIC	Questionnaire	Birth weight, visual sense, auditory sense, sleep on child's stomach?, child's bed or blanket soft?, child sleeps together in their parents' bed?, tummy time for baby?
		Physical examination	Whole body condition, head/fontanel, face, eye, nose, ear, oral cavity/neck, lung, heart, abdomen, genital, extremity, spine, nerve, skin
**Comorbidities**			
Hospital utilization within 6 months of age	NHIS		Visiting to pediatricians, Hospitalization, Visiting ER
Certain conditions originating in the perinatal period	NHIS (ICD-10 code)		
	P00-P04		Fetus and newborn affected by maternal factors and by complications of pregnancy, labor and delivery
	P05-P08		Disorders related to length of gestation and fetal growth
	P10-P15		Birth trauma
	P20-P29		Respiratory and cardiovascular disorders specific to the perinatal period
	P35-P39		Infections specific to the perinatal period
	P50-P61		Hemorrhagic and hematological disorders of fetus and newborn
	P70-P74		Transitory endocrine and metabolic disorders specific to fetus and newborn
	P80-P83		Conditions involving the integument and temperature regulation of fetus and newborn
	P90-P96		Other disorders originating in the perinatal period
Prevalent diseases diagnosed within 6 months of age	NHIS (ICD-10 code)		
	Z38.0		Singleton, born in hospital
	A08.4		Viral intestinal infection, unspecified
	A09.0		Other and unspecified gastroenteritis and colitis of infectious origin
	A09.9		Gastroenteritis and colitis of unspecified origin
	B37.0		Candidal stomatitis
	H10.0		Mucopurulent conjunctivitis
	H10.2		Other acute conjunctivitis
	H10.3		Acute conjunctivitis
	H10.9		Conjunctivitis, unspecified
	H60.9		Unspecified otitis externa
	H66.0		Acute suppurative otitis media
	H66.9		Otitis media, unspecified
	J00		Acute nasopharyngitis

	J01.9	Acute sinusitis, unspecified
	J02.9	Acute pharyngitis, unspecified
	J03.9	Acute tonsillitis, unspecified
	J06.0	Acute laryngopharyngitis
	J06.8	Other acute upper respiratory infections of multiple sites
	J06.9	Acute upper respiratory infection, unspecified
	J18.9	Pneumonia, unspecified
	J20.9	Acute bronchitis
	J21.9	Acute bronchiolitis
	J30.4	Allergic rhinitis
	J45.9	Other and unspecified asthma
	K21.9	Gastro-esophageal reflux disease without esophagitis
	K30	Functional dyspepsia
	K52.9	Noninfective gastroenteritis and colitis, unspecified
	K59.0	Constipation
	K59.1	Functional diarrhea
	K59.9	Functional intestinal disorder, unspecified
	L01.0	Impetigo [any organism] [any site]
	L20.8	Other atopic dermatitis
	L20.9	Atopic dermatitis
	L21.1	Seborrheic infantile dermatitis
	L21.9	Seborrheic dermatitis
	L22	Diaper dermatitis
	L23.9	Allergic contact dermatitis
	L24.9	Irritant contact dermatitis, unspecified cause
	L30.9	Dermatitis
	L98.0	Pyogenic granuloma
	N39.0	Urinary tract infection, site not specified
	P38	Omphalitis of newborn with or without mild haemorrhage
	P391	Neonatal conjunctivitis and dacryocystitis
	P599	Neonatal jaundice, unspecified
	P836	Umbilical polyp of newborn
	R11	Nausea and vomiting
	R50.9	Fever, unspecified
Drug used within 6 months of age	NHIS (drug classification code)	
	112	Sedative-hypnotics

	113	Antiepileptics
	114	Antipyretics
	117	Psyco-nervous system drug
	122	Muscle relaxant drug
	123	Autonomic nervous system drug
	124	Antispasmodics
	141	Antihistamine
	149	Other allergic drug
	211-219	Circulatory system drugs
	221-229	Respiratory system drugs
	232	Digestive anti-ulcer drug
	234	Antiacid drug
	237	Probiotics
	238	Laxative drug
	231, 233, 235, 236, 239	Other digestive system drugs
	241-244, 246-249	Hormone drugs
	245	Steroid

Table S3. Operating characteristics of the diagnostic codes used to define the diseases.

Diseases	Definition	N (%) in NICKs cohort (N = 917,707)	N (%) in this cohort (N = 146,706)
Main outcome			
IBS	Individuals with more than twice diagnosis ICD-10 code of IBS after the age of 4 years.	139,571 (15.2)	22,918 (15.6)
Sensitivity analysis			
IBS-1	Individuals with at least one diagnosis of ICD-10 code K58.X (IBS) and R10.X (abdominal pain) and at least one of ICD-10 code K59.1 (functional diarrhea), K52.2 (allergic and dietetic gastroenteritis and colitis), K52.8 (other specific noninfective gastroenteritis), or K59.0 (constipation) after the age of four years.	84,528 (9.2)	14,774 (10.0)
IBS-2	Individuals with two or more diagnoses of ICD-10 code K58.X (irritable bowel syndrome) with an interval of more than 6 months.	97,510 (10.6)	16,104 (10.9)

Table S4. Results of the questionnaires and physical examinations of the first round of the NHSPIC in the cohort

	Observed Data (N = 74,006)				Weighted Data (N = 146,706) [b]		
	N (%)[c]		Standardized Difference % [f]		N (%)[c]		Standardized Difference % [f]
	Tap water [d] (N = 28,850)	Purified water [e] (N = 45,156)			Tap water [d] (N = 28,850)	Purified water [e] (N = 45,156)	
**Questionnaire**							
Visual sense							
Appropriate	28,466 (98.7)	44,576 (98.7)	0.5		72,407 (98.7)	72,404 (98.7)	0.0
Suspected disease	382 (1.3)	569 (1.3)			948 (1.3)	946 (1.3)	
Auditory sense							
Appropriate	28,442 (98.7)	44,490 (98.7)	0.4		72,309 (98.6)	72,305 (98.6)	0.0
Suspected disease	382 (1.3)	569 (1.3)			1,046 (1.4)	1,045 (1.4)	
Sleep on child’s stomach?							
Yes	9,719 (33.7)	15,786 (35.0)	2.7		25,305 (34.5)	25,300 (34.5)	0.0
No	19,096 (66.3)	29,302 (65.0)			48,050 (35.5)	48,050 (65.5)	
Child’s bed or blanket soft?							
Yes	9,119 (31.6)	15,156 (33.6)	4.1		24,089 (32.8)	24,067 (32.8)	0.0
No	19,710 (68.4)	29,966 (66.4)			49,266 (67.2)	49,284 (67.2)	
Child sleeps together in their parents’ bed?							
Yes	16,125 (55.9)	25,375 (56.2)	0.6		41,133 (56.1)	41,123 (56.1)	0.0
No	12,704 (44.1)	19,746 (43.8)			32,222 (43.9)	32,228 (43.9)	
Tummy time for baby?							
Yes	26,109 (90.9)	41,373 (92.0)	3.9		67,171 (91.6)	67,165 (91.6)	0.0
No	2,621 (9.1)	3,618 (8.0)			6,185 (8.4)	6,186 (8.4)	
**Physical examination**							
Whole body condition							
Appropriate	28,835 (100.0)	45,136 (100.0)	0.8		73,347 (100.0)	73,342 (100.0)	0.0
Suspected abnormal	2 (0.0)	7 (0.0)			9 (0.0)	9 (0.0)	
Head/Fontanel							

Appropriate	28,816 (99.9)	45,119 (99.9)	0.7		73,310 (99.9)	73,305 (99.9)	0.0
Suspected abnormal	21 (0.1)	25 (0.1)			45 (0.1)	9 (0.0)	
Face							
Appropriate	28,832 (100.0)	45,137 (100.0)	0.1		73,343 (100.0)	73,339 (100.0)	0.0
Suspected abnormal	5 (0.0)	7 (0.0)			12 (0.0)	12 (0.0)	
Eye							
Appropriate	28,778 (99.8)	45,062 (99.8)	0.4		73,216 (99.8)	73,211 (99.8)	0.0
Suspected abnormal	59 (0.2)	82 (0.2)			139 (0.2)	139 (0.2)	
Nose							
Appropriate	28,810 (99.9)	45,092 (99.9)	0.6		73,275 (99.9)	73,272 (99.9)	0.0
Suspected abnormal	24 (0.1)	52 (0.1)			80 (0.1)	79 (0.1)	
Ear							
Appropriate	28,775 (99.8)	45,041 (99.8)	0.2		73,193 (99.8)	73,189 (99.8)	0.0
Suspected abnormal	62 (0.2)	103 (0.2)			163 (0.2)	162 (0.2)	
Oral cavity/Neck							
Appropriate	28,813 (99.9)	45,099 (99.9)	0.5		73,285 (99.9)	73,282 (99.9)	0.0
Suspected abnormal	24 (0.1)	45 (0.1)			70 (0.1)	69 (0.1)	
Lung							
Appropriate	28,811 (99.9)	45,118 (99.9)	1.2		73,303 (99.9)	73,298 (99.9)	0.0
Suspected abnormal	26 (0.1)	26 (0.1)			53 (0.1)	52 (0.1)	
Heart							
Appropriate	28,815 (99.9)	45,109 (99.9)	0.0		73,301 (99.9)	73,295 (99.9)	0.0
Suspected abnormal	22 (0.1)	35 (0.1)			55 (0.1)	56 (0.1)	
Abdomen							
Appropriate	28,817 (100.0)	45,128 (100.0)	0.0		73,329 (100.0)	73,325 (100.0)	0.0
Suspected abnormal	10 (0.0)	16 (0.0)			26 (0.0)	26 (0.0)	
Genital							
Appropriate	28,746 (99.7)	44,993 (99.7)	0.3		73,116 (99.7)	73,111 (99.7)	0.0

Suspected abnormal	91 (0.3)	151 (0.3)			239 (0.3)	240 (0.3)	
Extremity							
Appropriate	28,777 (99.8)	45,040 (99.8)	0.4		73,199 (99.8)	73,191 (99.8)	0.0
Suspected abnormal	60 (0.2)	104 (0.2)			157 (0.2)	160 (0.2)	
Spine							
Appropriate	28,830 (100.0)	45,138 (100.0)	0.8		73,342 (100.0)	73,338 (100.0)	0.0
Suspected abnormal	7 (0.0)	6 (0.0)			13 (0.0)	13 (0.0)	
Nerve							
Appropriate	28,883 (100.0)	45,137 (100.0)	0.1		73,345 (100.0)	73,340 (100.0)	0.0
Suspected abnormal	4 (0.0)	7 (0.0)			10 (0.0)	11 (0.0)	
Skin							
Appropriate	28,535 (99.0)	44,704 (99.0)	0.8		72,616 (99.0)	72,610 (99.0)	0.0
Suspected abnormal	302 (1.0)	440 (1.0)			739 (1.0)	741 (1.0)	

Abbreviations: NHSPIC , National Health Screening Program for Infants and Children; N, number

[a] Unless otherwise specified, all variables were assessed at the age of four to six months.

[b] Weighted using inverse probability of exposure weighting based on the propensity score. The propensity score was estimated using multivariable logistic regression with 103 previously covariates, as defined in eTable 2 in the Supplement. Participants in the reference group were weighted as (propensity score/[1-propensity score]). This method produces a weighted pseudo sample of participants in the reference group with the same distribution of measured covariates as the exposure group.

[c] Values are reported as N (%) unless otherwise indicated.

[d] The tap water (reference) group consisted of children who mainly consumed formula prepared with tap water during the first 4 to 6 months of life.

[e] The purified water group consisted of children who mainly consumed formula prepared with purified water during the first 4 to 6 months of life.

[f] The difference between the groups divided by the pooled standard deviation; a value greater than 10% is interpreted as a meaningful difference.

Table S5. Clinical characteristics of the participants [a]

Clinical Characteristics	Observed Data (N = 74,006)				Weighted Data (N = 146,706) [b]		
	N (%)[c]		Standardized Difference % [f]		N (%)[c]		Standardized Difference % [f]
	Tap water [d] (N = 28,850)	Purified water [e] (N = 45,156)			Tap water [d] (N = 28,850)	Purified water [e] (N = 45,156)	
Certain conditions (ICD-10 codes) originating in the perinatal period, No. (%)[c]							
Fetus and newborn affected by maternal factors and by complications of pregnancy, labour and delivery	591 (1.8)	851 (1.9)	0.6		1,350 (1.8)	1,355 (1.9)	0.0
Disorders related to length of gestation and fetal growth	99 (0.3)	176 (0.4)	0.8		269 (0.4)	270 (0.4)	0.0
Birth trauma	226 (0.8)	370 (0.8)	0.4		589 (0.8)	590 (0.8)	0.0
Respiratory and cardiovascular disorders specific to the perinatal period	1,199 (4.2)	1,793 (4.0)	0.9		2,974 (4.1)	2,967 (4.1)	0.0
Infections specific to the perinatal period	4,066 (14.1)	6,176 (13.7)	1.2		10,152 (13.8)	10,146 (13.8)	0.0
Haemorrhaggic and Haematological disorders of fetus and newborn	8,051 (27.9)	12,608 (27.9)	0.0		20,483 (27.9)	20,484 (27.9)	0.0
Transitory endocrine and metabolic disorders specific to fetus and newborn	721 (2.5)	1,174 (2.6)	0.6		1,877 (2.6)	1877 (2.6)	0.0
Conditions involving the integument and temperature regulation of fetus and newborn	1,166 (4.0)	1,601 (3.6)	2.6		2,746 (3.7)	2,748 (3.8)	0.0
Other disorders originating in the perinatal period	1,628 (5.6)	2,522 (5.6)	0.3		4,128 (5.6)	4,118 (5.6)	0.0
Prevalent diseases (ICD-10 codes) diagnosed within 6 months of age							
Singleton, born in hospital	245,264 (68.2)	5,911 (67.3)	1.9		247,791 (68.2)	246,112 (68.4)	0.1
Viral intestinal infection, unspecified	9,741 (2.7)	459 (5.2)	12.9		10,064 (2.8)	11,772 (3.3)	0.6
Other and unspecified gastroenteritis and colitis of infectious origin	18,362 (5.1)	707 (8.0)	11.9		18,802 (5.2)	20,941 (5.8)	0.7
Gastroenteritis and colitis of unspecified origin	20,055 (5.6)	781 (8.9)	12.8		20,541 (5.7)	22,505 (6.3)	0.8
Candidal stomatitis	6,325 (1.8)	169 (1.9)	1.2		6,402 (1.8)	6,768 (1.9)	0.3
Mucopurulent conjunctivitis	5,620 (1.6)	133 (1.5)	0.4		5,674 (1.6)	6,389 (1.8)	0.2
Other acute conjunctivitis	6,863 (1.9)	170 (1.9)	0.2		6,934 (1.9)	7,539 (2.1)	0.0

acute conjunctivitis	13,848 (3.8)	335 (3.8)	0.2		14,011 (3.9)	14,418 (4.0)	0.4
Conjunctivitis, unspecified	14,776 (4.1)	397 (4.5)	2.0		14,961 (4.1)	14,662 (4.1)	0.4
Unspecified otitis externa	4,455 (1.2)	137 (1.6)	2.7		4,511 (1.2)	4,579 (1.3)	0.1
Acute suppurative otitis media	14,875 (4.1)	349 (4.0)	0.8		15,000 (4.1)	14,632 (4.1)	0.4
Otitis media, unspecified	7,989 (2.2)	204 (2.3)	0.7		8,058 (2.2)	7,869 (2.2)	0.2
Acute nasopharyngitis	104,381 (29.0)	3,056 (34.8)	12.4		105,974 (29.2)	107,273 (29.8)	0.1
Acute sinusitis, unspecified	12,585 (3.5)	304 (3.5)	0.2		12,707 (3.5)	12,261 (3.4)	0.4
Acute pharyngitis	37,920 (10.5)	1,103 (12.6)	6.3		38,483 (10.6)	39,901 (11.1)	0.6
Acute tonsillitis, unspecified	18,232 (5.1)	464 (5.3)	1.0		18,411 (5.1)	18,064 (5.0)	0.1
Acute laryngopharyngitis	9,189 (2.6)	229 (2.6)	0.3		9,276 (2.6)	9,299 (2.6)	0.0
Other acute upper respiratory infections of multiple sites	9,292 (2.6)	265 (3.0)	2.6		9,427 (2.6)	9,916 (2.8)	0.3
Acute upper respiratory infection, unspecified	81,665 (22.7)	2,198 (25.0)	5.4		82,711 (22.8)	84,377 (23.4)	0.5
Pneumonia, unspecified	9,496 (2.6)	225 (2.6)	0.5		9,569 (2.6)	10,044 (2.8)	0.6
Acute bronchitis	66,304 (18.4)	1,634 (18.6)	0.4		66,995 (18.4)	66,559 (18.5)	0.7
Acute bronchiolitis	48,798 (13.6)	1,129 (12.8)	2.1		49,176 (13.5)	50,601 (14.1)	0.7
Allergic rhinitis	8,759 (2.4)	208 (2.4)	0.4		8,853 (2.4)	8,289 (2.3)	0.7
Other and unspecified asthma	8,334 (2.3)	181 (2.1)	1.8		8,384 (2.3)	8,510 (2.4)	0.2
Gastro-esophageal reflux disease without esophagitis	8,416 (2.3)	333 (3.8)	8.4		8,617 (2.4)	8,590 (2.3)	0.4
Functional dyspepsia	7,635 (2.1)	315 (3.6)	8.8		7,834 (2.2)	8,289 (5.1)	0.2
Noninfective gastroenteritis and colitis, unspecified	16,372 (4.6)	561 (6.4)	8.1		16,709 (4.6)	18,306 (5.1)	0.8
Constipation	15,365 (4.3)	574 (6.5)	10.0		15,699 (4.3)	16,272 (4.5)	0.7
Functional diarrhea	5,223 (1.5)	205 (2.3)	6.5		5,360 (1.5)	5,375 (1.5)	0.0
Functional intestinal disorder, unspecified	10,060 (2.8)	357 (4.1)	7.0		10,248 (2.8)	10,274 (2.9)	0.1
Impetigo [any organism] [any site]	5,586 (1.6)	146 (1.7)	0.9		5,641 (1.6)	5,642 (1.6)	0.0
Other atopic dermatitis	20,095 (5.6)	479 (5.5)	0.6		20,245 (5.6)	19,907 (5.5)	0.3
Atopic dermatitis	57,318 (15.9)	1,507 (17.2)	3.3		58,007 (16.0)	56,019 (15.6)	0.0

Seborrhoeic infantile dermatitis	9,003 (2.5)	253 (2.9)	2.3		9,124 (2.5)	9,301 (2.6)	0.6
Seoborrhoeic dermatitis	7,460 (2.1)	199 (2.3)	1.3		7,563 (2.1)	7,599 (2.1)	0.2
Diaper dermatitis	27,123 (7.5)	784 (8.9)	5.0		27,525 (7.6)	27,099 (7.5)	0.4
Allergic contact dermatitis	16,542 (4.6)	479 (5.5)	3.9		16,794 (4.6)	17,709 (4.9)	0.1
Irritant contact dermatitis, unspecified cause	6,867 (1.9)	220 (2.5)	4.1		6,974 (1.9)	7,213 (2.0)	0.2
Dermatitis	15,342 (4.3)	480 (5.5)	5.6		15,630 (4.3)	15,785 (4.4)	0.1
Pyogenic granuloma	4,750 (1.3)	131 (1.5)	1.4		4,820 (1.3)	4,498 (1.3)	0.0
Urinary tract infection, site not specified	7,731 (2.1)	231 (2.6)	3.1		7,861 (2.2)	8,302 (2.3)	0.1
Omphalitis of newborn with or without mild haemorrhage	20,223 (5.6)	559 (6.4)	3.1		20,489 (5.6)	20,714 (5.8)	0.5
Neonatal conjunctivitis and dacryocystitis	10,521 (2.9)	293 (3.3)	2.4		10,654 (2.9)	10,584 (2.9)	0.3
Neonatal jaundice, unspecified	86,648 (24.1)	2,117 (24.1)	0.0		87,572 (24.1)	86,745 (24.1)	0.5
Umbilical polyp of newborn	6,005 (1.7)	125 (1.4)	2.0		6,053 (1.7)	6,044 (1.7)	0.7
Nausea and vomiting	10,334 (2.9)	495 (5.6)	13.7		10,699 (2.9)	11,869 (3.3)	0.5
Fever, unspecified	18,237 (5.1)	524 (6.0)	3.9		18,507 (5.1)	21,194 (5.9)	0.1
Drug (drug classification code) used within 6 months of age							
Sedative-hypnotics	1,767 (6.1)	2,838 (6.3)	0.7		4,566 (6.2)	4,559 (6.2)	0.0
Antiepileptics	20 (0.1)	22 (0.1)	0.8		43 (0.1)	43 (0.1)	0.0
Antipyretics	17,166 (59.5)	28,027 (62.1)	5.3		44,808 (61.1)	44,796 (61.1)	0.0
Psyco-nervous system drug	3,577 (12.4)	6,343 (14.1)	4.9		9,851 (13.4)	9,837 (13.4)	0.0
Muscle relaxant drug	70 (0.2)	123 (0.3)	0.6		193 (0.3)	192 (0.3)	0.0
Autonomic nervous system drug	94 (0.3)	177 (0.4)	1.1		267 (0.4)	268 (0.4)	0.0
Antispasmodics	1,519 (5.3)	2,498 (5.5)	1.2		3,983 (5.4)	3,977 (5.4)	0.0
Antihistamine	19,707 (68.3)	32,541 (72.1)	8.2		51,843 (70.7)	51,817 (70.6)	0.0
Other allergic drug	655 (2.3)	1,118 (2.5)	1.4		1,746 (2.4)	1,752 (2.4)	0.0
Circulatory system drugs	475 (1.7)	860 (1.9)	2.0		1,331 (1.8)	1,326 (1.8)	0.0
Respiratory system drugs	21,964 (76.1)	35,691 (79.0)	7.0		57,195 (78.0)	57,169 (77.9)	0.0
Digestive anti-ulcer drug	524 (1.8)	884 (2.0)	1.0		1,384 (1.9)	1,390 (1.9)	0.0

Antiacid drug	313 (1.1)	558 (1.2)	1.4		858 (1.2)	861 (1.2)	0.0
Probiotics	20,061 (69.5)	32,396 (71.7)	4.8		51,976 (70.9)	51,983 (70.2)	0.0
Laxative drug	39 (0.1)	76 (0.2)	0.9		114 (0.2)	114 (0.2)	0.0
Other digestive system drugs	11,080 (38.4)	17,939 (39.7)	2.7		28,760 (39.2)	28,760 (39.2)	0.0
Hormone drugs	3,501 (12.1)	6,231 (13.8)	5.0		9,638 (13.1)	9,643 (13.2)	0.0
Steroid	3,481 (12.1)	6,199 (13.7)	5.0		9,588 (13.1)	9,592 (13.1)	0.0

Abbreviations: N, number; ICD, International Classification of Diseases; ER, emergency room; ICU, intensive care unit.

[a] Unless otherwise specified, all baseline characteristics were assessed in the first 6 months of age of the participants.

[b] Weighted using inverse probability of exposure weighting based on the propensity score. The propensity score was estimated using multivariable logistic regression with 103 previously covariates, as defined in eTable 2 in the Supplement. Participants in the reference group were weighted as (propensity score/[1-propensity score]). This method produces a weighted pseudo sample of participants in the reference group with the same distribution of measured covariates as the exposure group.

[c] Results are reported as N (%) unless otherwise indicated.

[d] The tap water (reference) group consisted of children who mainly consumed formula prepared with tap water during the first 4 to 6 months of life.

[e] The purified water group consisted of children who mainly consumed formula prepared with purified water during the first 4 to 6 months of life.

[f] Differences greater than 10% were interpreted as meaningful differences. All the standardized differences in the cohort values were < 0.05.

Table S6. Post-hoc analysis using a negative control exposure to assess the risk of IBS between tap water and commercial bottled water

		Weighted Data (N = 146,706) [a]		HR (95% CI) [b]
		Number of participants	No. of IBS [e] (%)	
Exposure	Commercial bottled water [c]	49,024	7,526 (15.4)	1.004 (0.960 to 1.051)
Referent	Tap water [d]	49,040	7,490 (15.3)	

[a] Weighted using inverse probability of exposure weighting based on the propensity score. The propensity score was estimated using multivariable logistic regression with 103 previously covariates, as defined in eTable 2 in the Supplement. Participants in the reference group were weighted as (propensity score/[1-propensity score]). This method produces a weighted pseudo sample of participants in the reference group with the same distribution of measured covariates as the exposure group.

[b] The hazard ratios were assessed using a Cox proportional hazards model to assess the risk of IBS according to type of used water (tap water vs. purified water).

[c] The commercial bottle water group consists of children who have mainly used the commercial bottled water during the first 4 to 6 months of age.

[d] The tap water (reference) group consisted of children who mainly consumed formula prepared with tap water during the first 4 to 6 months of life.

[e] IBS was defined as having the diagnosis of ICD-10 code K58.X (irritable bowel syndrome) more than twice, after four years of age.
